# A protocol for randomized controlled trial on multidisciplinary interventions for mobility limitation in the older adults (M-MobiLE)

**DOI:** 10.1186/s12877-023-04117-4

**Published:** 2023-08-08

**Authors:** Guanzhen Wang, Li Zhang, Tong Ji, Wanshu Zhang, Linlin Peng, Shanshan Shen, Xiaolei Liu, Yanqing Shi, Xujiao Chen, Qiong Chen, Yun Li, Lina Ma, the M-MobiLE Study Working Group

**Affiliations:** 1 Department of Geriatrics, National Clinical Research Center for Geriatric Disorders, Xuanwu Hospital Capital Medical University, Beijing, China; 2 Department of Geriatrics, Xiangya Hospital Central South University, Changsha, China; 3 National Clinical Research Center for Geriatric Disorders, Xiangya Hospital, Central South University, Changsha, China; 4 Department of Geriatrics, Zhejiang hospital, Hangzhou, China; 5 Department of Geriatrics, West China Hospital Sichuan University, Chengdu, China; 6 Department of Geriatrics, Fujian Medical University Union Hospital, Fuzhou, China

**Keywords:** Mobility limitation, Multidisciplinary team, Treatment, Randomized controlled study, Older adults

## Abstract

**Background:**

Mobility limitation—the loss of exercise capacity or independent living ability—is a common geriatric syndrome in older adults. As a potentially reversible precursor to disability, mobility limitation is influenced by various factors. Moreover, its complex physiological mechanism hinders good therapeutic outcomes with a single-factor intervention. Most hospitals have not incorporated the diagnosis and evaluation of mobility limitation into medical routines nor developed a multidisciplinary team (MDT) treatment plan. We aim to conduct a clinical trial titled “A Multidisciplinary-team approach for management of Mobility Limitation in Elderly (M-MobiLE)” to explore the effect of the MDT decision-making intervention for mobility limitation.

**Methods:**

The M-MobiLE study will be a multicenter, randomized, and controlled trial. We will recruit a minimum of 66 older inpatients with mobility limitation from at least five hospitals. Older patients with mobility limitation admitted to the geriatrics department will be included. Short-Physical Performance Battery (SPPB), Activities of Daily Living (ADL), Function Impairment Screening Tool (FIST), Geriatric Depression Scale (GDS-15), Short Form − 12 (SF-12), Fried frailty phenotype, social frailty, Morse Fall Risk Scale, SARC-CalF, Mini-Mental State Examination (MMSE), Mini-Nutritional Assessment Short-Form (MNA-SF), and intrinsic capacity will be assessed. The intervention group will receive an exercise-centered individualized MDT treatment, including exercise, educational, nutritional, medical, and comorbidity interventions; the control group will receive standard medical treatment. The primary outcome is the change in the SPPB score, and the secondary outcomes include increased SF-12, ADL, FIST, MMSE, MNA-SF, and intrinsic capacity scores and decreased GDS-15 and SARC-CalF scores.

**Conclusion:**

Our results will help develop a multidisciplinary decision-making clinical pathway for inpatients with mobility limitation, which can be used to identify patients with mobility limitation more effectively, improve mobility, and reduce the risk of falls, frailty, and death in older inpatients. The implementation of this MDT strategy may standardize the treatment of mobility limitation, reduce adverse prognosis, and improve quality of life.

**Trial registration:**

ChiCTR, ChiCTR2200056756, Registered 19 February 2022.

## Introduction

The proportion of older adults is increasing in society. Safeguarding healthy aging in older adults has significant implications for individuals and society. Mobility limitation—a type of geriatric syndrome —refers to the loss of exercise capacity or independent living ability [1]. It is often the first noticeable sign of functional decline [2, 3] and is diagnosed by assessing a person’s ability to move from a bed or chair, walk a quarter of a mile, or climb stairs independently, or by assessing distance walked from the home [1, 3–6]. Studies have shown that approximately one-third of geriatric patients experience mobility limitation [7]. Notably, hospitalized patients are particularly affected, with the incidence of loss of movement being as high as 17% due to the limitation of movement space and the presence of diseases [8]. Additionally, weekly bed rest may result in losing 5–10% of muscle strength in older adults during hospitalization [9, 10].

The effect of mobility limitation on older adults is multi-dimensional. Mobility limitation was significantly associated with depressive symptoms [11], decreased quality of life [12], increased disability [13], falls [14], and mortality [15]. Signs of mobility limitation can be used as indicators to predict physical health impairment, loss of independent living ability, hospitalization, and death. Additionally, mobility limitation is a huge hidden danger affecting the health and safety of older adults. It is affected by different factors, including nutrition [16, 17], psychology [11], obesity [18, 19], sedentary lifestyle [14, 20], muscle dysfunction, joint damage [21, 22], and cardiovascular [23, 24], respiratory [24, 25], and endocrine diseases [26–28].

Mobility limitation has received widespread attention due to increased awareness of healthy aging. Some studies have achieved remarkable results with the intervention of single factor, such as testosterone [26, 29], protein [17], blood pressure [30], and resistance exercise [31, 32]. Therefore, the feasibility and necessity of mobility limitation intervention have been clarified.

However, since mobility limitation indicates a decline in the body’s comprehensive reserve capacity [3], many influencing factors co-exist; it is difficult to use a single indicator for its intervention. Additionally, treatment with a single factor cannot remove the root cause in all patients. Mobility limitation is often accompanied by comorbidities or functional decline; therefore, older adults’ physical, psychological, and social functions should be evaluated from multiple dimensions. It is crucial to comprehensively assess the influencing factors of mobility limitation and administer individualized multidisciplinary team (MDT) treatment. However, the diagnosis and treatment of mobility limitation are not currently integrated into routine medical care, and there are no studies on using the MDT approach for treating mobility limitation in a hospital setting [1, 14].

This study aims to conduct a clinical trial called " A Multidisciplinary-team approach for management of Mobility Limitation in Elderly (M-MobiLE)” in a hospital setting to determine the effect of MDT in managing mobility limitation. We hypothesize that MDT can improve mobility (Short-Physical Performance Battery [SPPB]) in older adults with mobility limitation. Additionally, we hypothesize that MDT can improve intrinsic capacity, quality of life, depression, cognition, frailty, and nutritional status, reduce the incidence of frailty, falls, and other adverse events, shorten hospital stay, and reduce mortality.

## Method and design

### Study design and setting

**Fig. 1:**
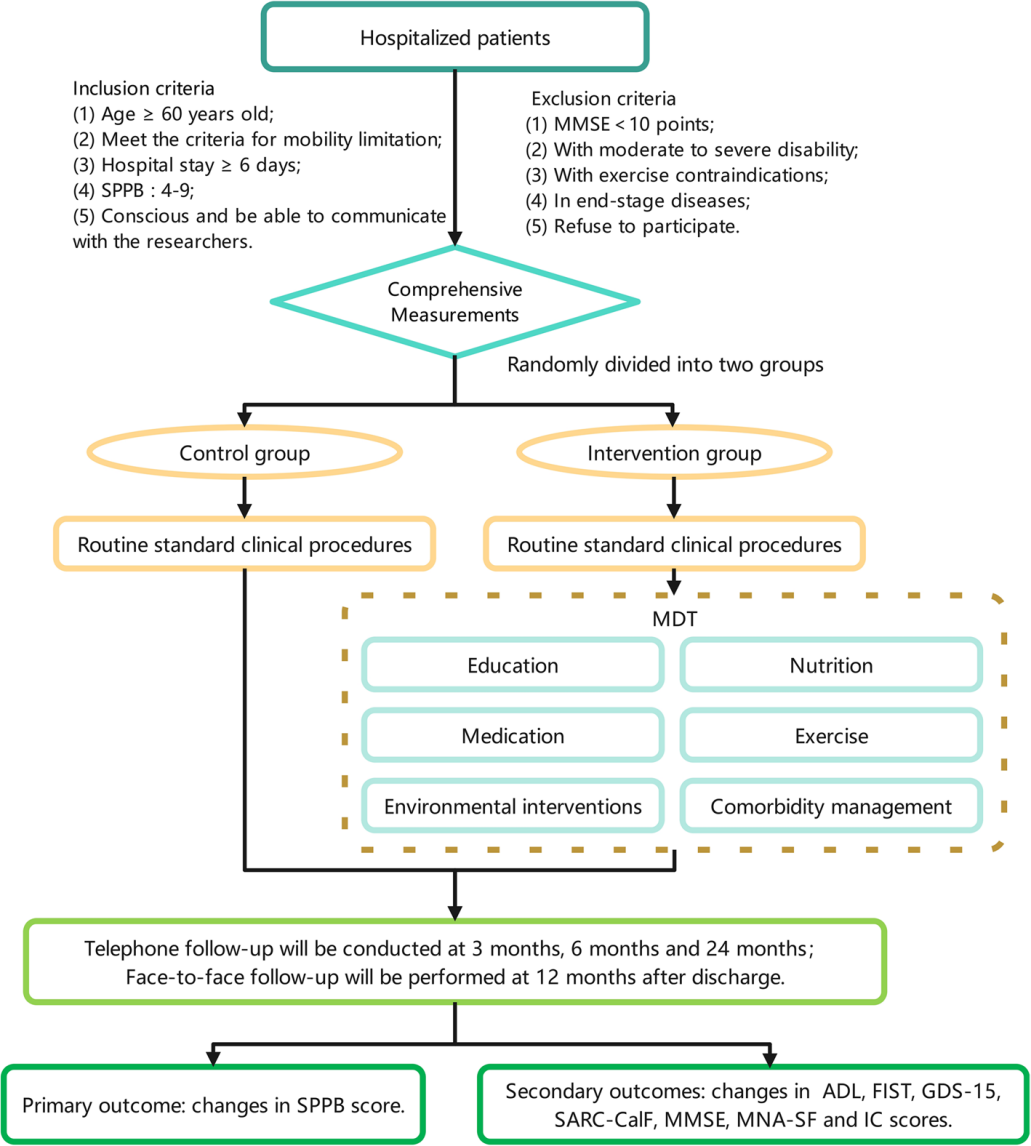
Flow chart of the M-MobiLE study. SPPB, Short-Physical Performance Battery; MDT, multidisciplinary team; ADL, Activities of Daily Living; GDS-15, Geriatric Depression Scale; MMSE, Mini-Mental State Examination; MNA-SF, Mini Nutritional Assessment short-form; IC, Intrinsic Capacity

We will conduct a prospective, multicenter, randomized, parallel, and controlled trial in the geriatric departments of at least five hospitals. A two-arm follow-up cohort will be established in this study. The control group will be placed on routine treatments, and the intervention group will be treated with an MDT approach, including exercise, educational, nutritional, medical, comorbidity, and environmental interventions. The multidisciplinary consultation team will include geriatricians, rehabilitation physicians, nutritionists, nurses, pharmacists, ophthalmologists, otolaryngologists, neurologists, cardiologist, rheumatologists, endocrinologists, psychologists, and pain management physicians. Furthermore, we will develop an individualized exercise prescription based on the “International Exercise Recommendations in Adults (ICFSR): Expert consensus guidelines” and the recommendations of the rehabilitation physician. The assessment for mobility limitation will be completed within 48h after admission, followed by MDT. Guidance recommendations for comprehensive interventions will be extended to 2 years after discharge, and follow-up assessments will be performed at 3, 6, 12, and 24 months after discharge (Fig. 1).

#### Inclusion criteria

Mobility limitation will be screened by asking the patient to answer subjective questions or through a simple physical fitness test [1, 3, 33, 34]. We have integrated both methods to ensure the accuracy. The following conditions will be used for screening: (1) compared with the past, it is now more difficult to climb 10 steps in a row or walk 400 m (including changes in style or speed reduction), (2) five time-chair sit-to-stand ≥ 14 s, and (3) timed Up and Go ≥ 10 s.

Inpatients who meet the following criteria will be included in the study: (1) age ≥ 60 years, (2) meet the criteria for mobility limitation, (3) hospital stay ≥ 6 days, (4) SPPB ranges from 4 to 9 (Mobility limitation is defined as SPPB≤9 points [33, 35]. However, in order to ensure the safety of exercise in the hospital, we will exclude patients with a score of 4 or less.), and (5) conscious and able to communicate with the researcher.

#### Exclusion criteria

Patients who meet the following criteria will be excluded: (1) Mini-Mental State Examination (MMSE) < 10 points, (2) moderate to severe disability, (3) exercise contraindications^*^, (4) end-stage diseases, and (5) refusal to participate. Additionally, participants will be considered lost to follow-up if they miss any follow-up or if necessary clinical data cannot be obtained during the follow-up period.

^*^Exercise contraindications [36, 37]: acute uncompensated diabetes or poorly controlled hypoglycemia; dissecting aortic aneurysm; acute myocardial infarction or recent unstable angina; acute or severe heart failure; poorly controlled atrial or ventricular arrhythmias; severe aortic stenosis; endocarditis or acute pericarditis; poorly controlled high blood pressure; poorly controlled postural hypotension; acute thromboembolism; acute or severe respiratory failure; and fracture within the past 1 month.

### Randomization

Older adults who meet the inclusion criteria in the geriatric department will be numbered according to the time of admission. Moreover, we will use R and adopt a block randomization method to randomly assign patients to the control and intervention groups in a 1:1 ratio. This study will be open-label.

### Measurements

#### Assessment

**Fig. 2:**
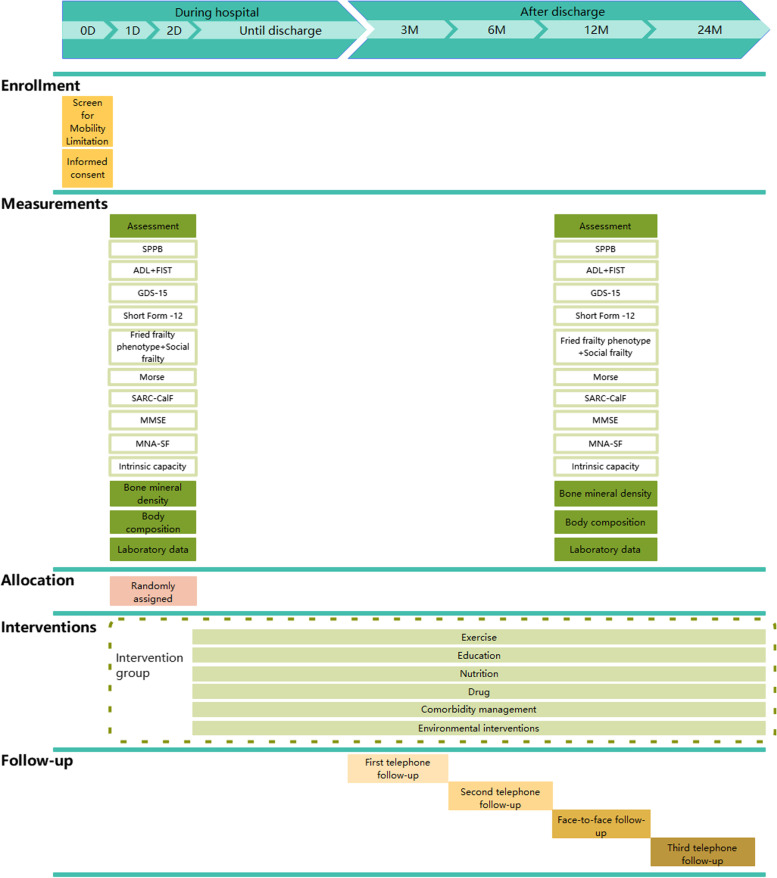
Schedule of enrollment, measurement, allocation, intervention, and follow-up. SPPB, Short-Physical Performance Battery; ADL, Activities of daily living; GDS-15, Geriatric Depression Scale; SF-12, The Short Form-12; MMSE, Mini-Mental State Examination; MNA-SF, Mini Nutritional Assessment short-form; IC, Intrinsic Capacity

The initial assessment will be completed within 48 h of admission. Subsequently, follow-up by telephone will be performed at 3, 6, and 24 months after discharge. Additionally, a face-to-face follow-up will be conducted at 12 months after discharge (Fig. 2). SPPB will be used to assess mobility. SPPB is a well-established tool for assessing lower extremity physical performance status. It includes balance, pace, and standing-sitting tests, with a total score of 0–12, with lower scores indicating poorer exercise capacity [38]. The activities of daily living (ADL) and the functional impairment screening tool (FIST) scales will be used to assess physical function. The FIST assessment entails 16 questions with a total score of 16. The higher the score, the better the performance [39]. We will use Geriatric Depression Scale (GDS) -15 to assess depression. It has a total score of 15 points, with 5–9 indicating that the patient may be prone to depression and ≥ 10 indicating depression [40, 41]. The Short Form-12 will be used to assess the quality of life [42]. Fried frailty phenotype includes unintentional weight loss, exhaustion, weakness, slowness, and low physical activity. A score of 0 indicates robustness, 1–2 indicates pre-frail, and ≥ 3 indicates frailty [43]. Moreover, we will evaluate social frailty using the Help, Participation, Loneliness, Financial, and Talk (HALFT) scale, and those who score ≥ 3 will be classified as socially frailty, 1–2 as pre-social frailty, and 0 will be considered normal [44]. Additionally, the Morse Fall Risk Scale will be used to assess falls. Scores ≤ 23, 24–44, and ≥ 45 are classified as low intermediate and high risks, respectively [45].

Furthermore, we will assess sarcopenia using SARC-CalF, which includes six questions: lifting 10 pounds, walking from one end of the room to the other, moving from a chair to a bed, climbing one flight (10 steps) of stairs, number of falls in the past 1 year, and calf circumference. Patients with a score ≥ 11 will be considered to have a high risk of sarcopenia [46, 47]. MMSE will be used to assess cognition. Its scores range from 0 to 30, with 21–26, 10–20, and 0–9 indicating mild, moderate, and severe cognitive impairments, respectively [48, 49]. Nutritional status will be assessed using the Mini Nutritional Assessment Short-Form (MNA-SF) [50–52]. A score of 0–7 means the patient is malnourished, 8–11 means risk of malnutrition, and 12–14 indicates normal nutritional status. Intrinsic capacity includes five aspects: cognitive decline, limited mobility, malnutrition, sensory loss, and depressive symptoms [53].

#### Bone mineral density

We will use an X-ray osteodensitometer (LUNAR iDXA, GE Healthcare, Madison, WI, USA) to measure bone mineral density, T-score, and Z-score of the lumbar spine and femoral bone in older adults.

#### Body composition

We will use a body composition meter (JAWON-IOI353, Jawon Medical, Gyeongsan, Korea) to measure the body fat mass, soft lean mass, and basal metabolic rate of older adults through bioelectrical impedance analysis.

#### Laboratory data

We will measure the following parameters: leukocyte counts, hemoglobin concentration, blood biochemistry, coagulation, glycated hemoglobin, brain natriuretic peptide, C-reactive protein, interleukin-6, vitamin D, and ferritin.

### Intervention scheme

**Fig. 3:**
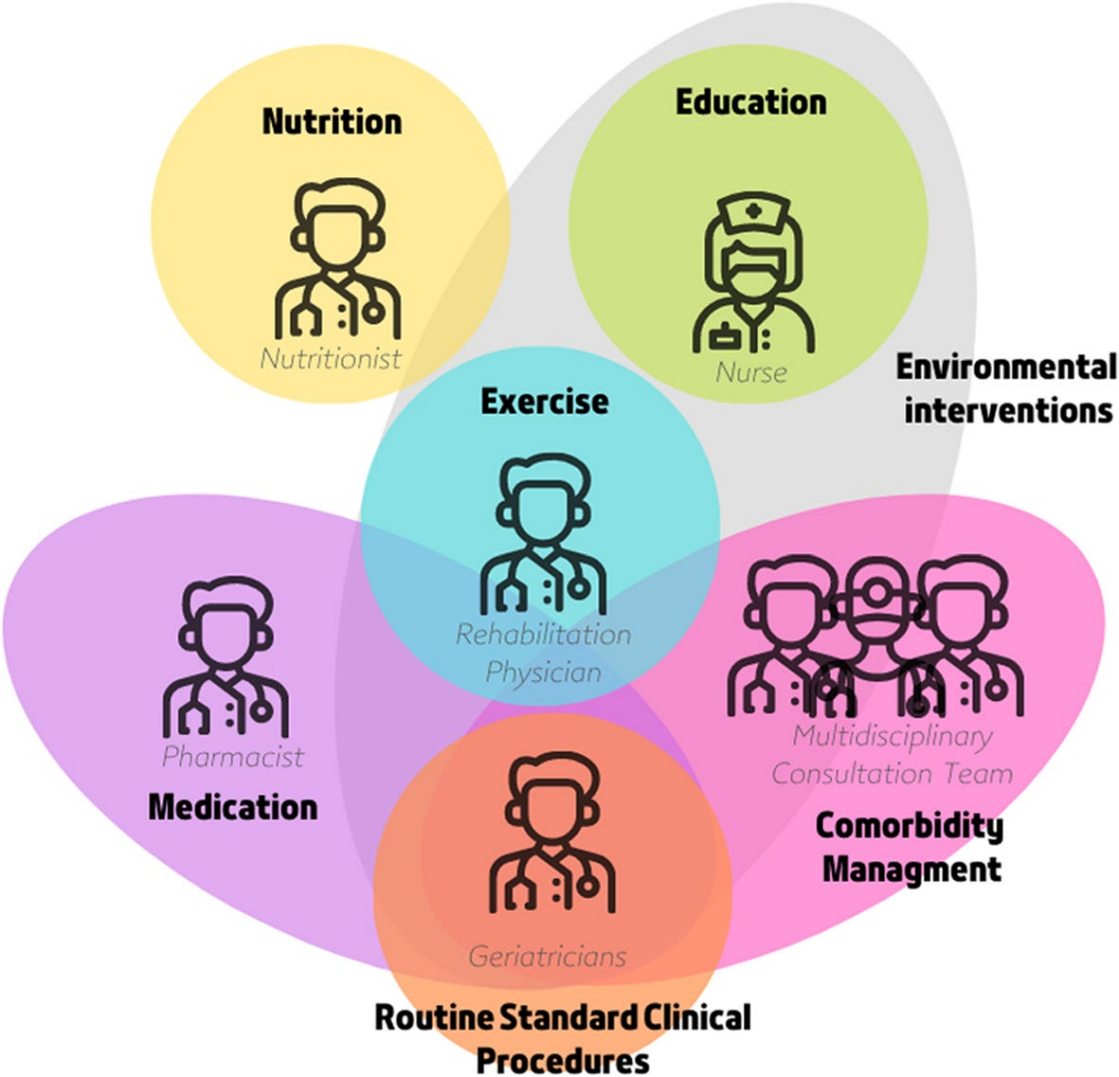
Multidisciplinary team (MDT) intervention program. MDT is based on routine medical procedures with exercise intervention as the core. The multidisciplinary consultation team includes ophthalmologists, otolaryngologists, neurologists, cardiologists, rheumatologists and immunologists, endocrinologists, psychologists, and pain management physicians

Patients with mobility limitation will be randomly divided into control (routine standard clinical procedures) and intervention groups (MDT + routine standard clinical procedures) (Fig. 3).

#### Exercise intervention

**Fig. 4:**
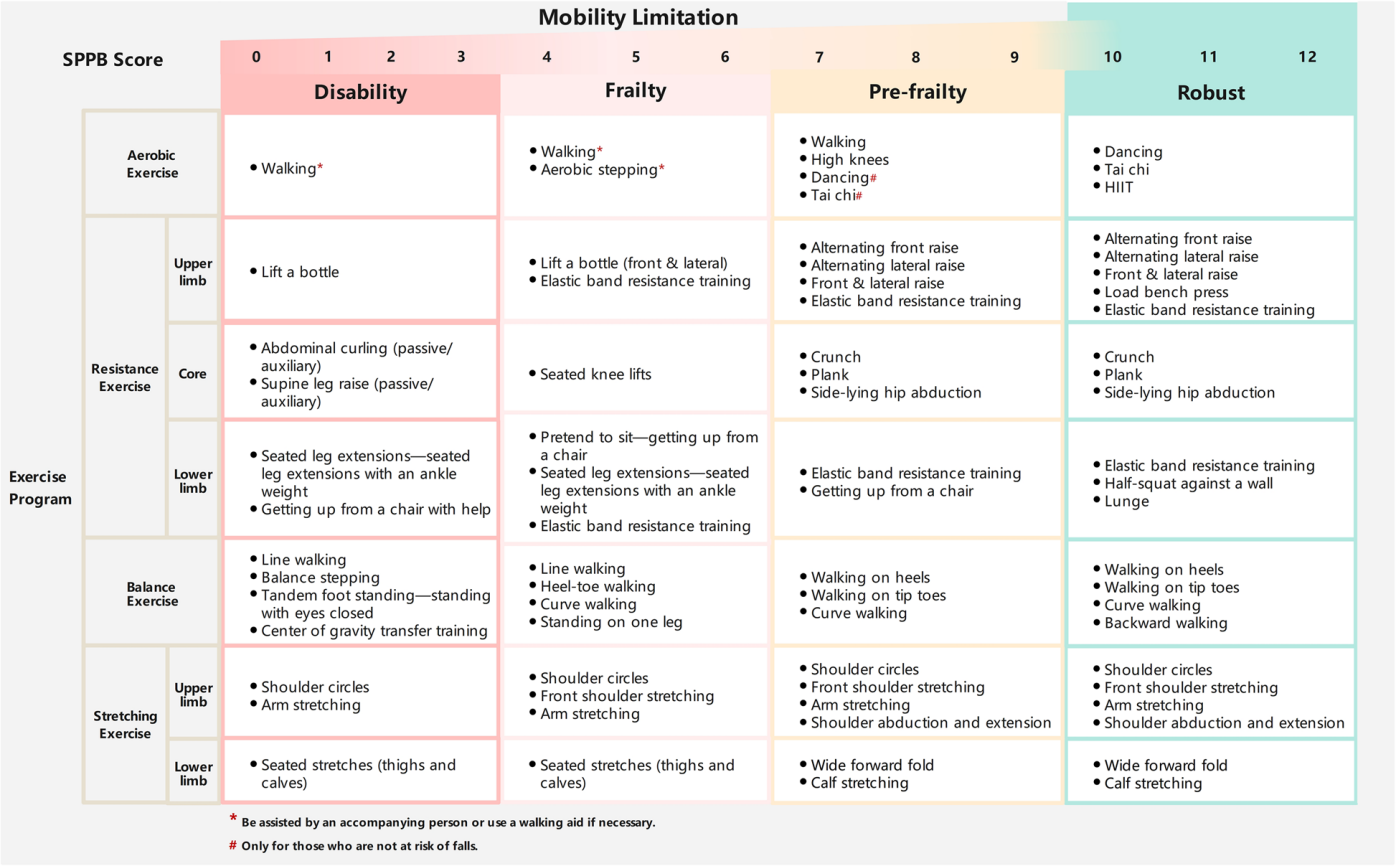
The M-MobiLE exercise intervention program includes aerobic, resistance, balance, and stretching exercises. An individualized exercise program will be developed for the patient based on SPPB scores

The exercise intervention plan will be based on the ICFSR (2021) recommendation [54], using the M-MobiLE exercise program and adjusting the exercise program and frequency guided by a rehabilitation physician. The M-Mobile exercise intervention plan includes aerobic, resistance, balance, and stretching exercises (Fig. 4).

Aerobic exercises will be conducted for 30–60 min daily, and the exercise duration can be gradually increased. Patients will be asked to complete at least 150 min of aerobic exercise per week, at least 5 days a week, at an intensity such that the patient has mild dyspnea when active but can still talk easily. Resistance exercises will be performed progressively, gradually increasing the amount of exercise. Patients will perform muscle-strengthening activities at least two days a week. Balance exercise will be based on the actual situation of older adults to choose a suitable method and prevent injury. Patients can borrow a stable chair for assistance at the beginning of the exercise or exercise accompanied by others. Stretching exercises will be performed at least twice a week. The specific amount of exercise will be determined during the training by a rehabilitation physician to ensure exercise safety. During hospitalization, patients in the intervention group will be trained 3–5 times weekly. After the exercise, patients will record their experienceit in the exercise manual: easy, difficult, or very difficult. Additionally, patients will be instructed to keep recording in their exercise diaries after discharge, filling in the number of completed weekly sessions and training experience until the last follow-up.

#### Educational interventions

The nursing team will distribute mobility limitation treatment brochures to patients and instruct patients who can get out of bed to avoid prolonged bed rest during hospitalization.

#### Nutritional interventions

Nutritional interventions will be determined based on the MNA-SF scores and nutritionist's recommendations.

#### Pharmacological interventions

If a medication significantly increases the risk of mobility limitation, the pharmacist will help geriatrician consider whether to discontinue, switch, or reduce the dose.

#### Comorbidity management

Geriatricians will cooperate with other multidisciplinary consultation teams to diagnose and intervene in the risk factors associated with mobility limitation. The multidisciplinary consultation team will include ophthalmologists, otolaryngologists, neurologists, cardiologists, rheumatologists and immunologists, endocrinologists, and pain management physicians. Additionally, patients with psychological diseases will be transferred to the psychology department for further treatment.

#### Environmental interventions

Geriatricians, rehabilitation physicians, and nursing teams will educate patients and their families on reasonably optimizing the home environment to avoid adverse events, such as falls.

### Study outcomes

The core of this study is to explore the effect of MDT on mobility in older adults with mobility limitation. The primary outcome is a change in the SPPB score, and secondary outcomes include changes in ADL, FIST, GDS-15, SARC-CalF, MMSE, MNA-SF, and intrinsic capacity scores.

### Sample size

This will be a parallel randomized-controlled study, and the ratio of the intervention to the control group will be 1:1. A randomized-controlled trial (RCT) in 2021 [55] reported an SPPB of 6.1 points in the control group, and the mean difference between both groups after exercise intervention was 2.4 [56]. Our sample estimate is a one-sided test with a first-class error of 0.025 and a power of 90%. The sample size N1 = N2 = 26 was calculated using PASS 15. Assuming that the rate of loss to follow-up is 20%, the minimum sample size will be N1 = N2 = 26/0.8 = 33. Therefore, a minimum of 66 patients will be recruited for this study.

### Data collection, validation, and management

The medical data will be collected using the Hospital Information System. Additionally, data input will be done by trained staff using Epidata. The database will be checked by the data administrator, who will desensitize, structure, standardize, and control the data, design the corresponding database system project according to the research plan, and set logical check conditions for entry.

### Statistical analysis

Baseline characteristics will be described using statistics, such as mean ± SD, mean rank, or number and percentage. Differences in the characteristics between both groups of outcomes will be assessed using chi-square/Fisher’s exact test for categorical variables and t-test/Wilcoxon-rank sum test for continuous variables. Potential confounding factors will be analyzed using univariate and multivariate logistic regression analyses. The statistical significance will be set at *p* < 0.05 (two-tailed), and statistical analyses will be performed using R.

## Discussion

As a geriatric syndrome with an extremely high prevalence, the diagnosis, evaluation, and treatment of mobility limitation are critical. Besides influencing various diseases, such as heart disease, respiratory disease, depression, and cognitive impairment [11, 23–25], mobility limitation increases the risk of falls, fractures, and death [14, 15]. As research on mobility limitation increases, so has the discovery of its influencing factors. These risk factors, including obesity, malnutrition, and low physical activity, can be intervened to prevent, alleviate, and treat mobility limitation [16–20]. Therefore, the complex factors that influence mobility limitation should be identified, and a comprehensive and individualized MDT program to reduce its prevalence should be formulated.

Many patients with mobility limitation have other comorbid diseases or functional impairments, and these factors are often mutually causal with mobility limitation [1]. Furthermore, although treating mobility limitation from a single angle may be effective, this approach is not comprehensive, limiting the treatment effect. Similar to the barrel effect, it may manifest as a mobility limitation if comorbidity is not addressed. Therefore, effectively managing or alleviating mobility limitation presents a challenge for geriatric doctors and nurses.

Comprehensive geriatric assessment can identify the risk factors associated with mobility limitation and reveal functional impairment in patients with mobility limitation [57, 58]. Based on this assessment, individualized routine exercise and educational, nutritional, medical, and comorbidity interventions can comprehensively eliminate or alleviate the causes of mobility limitation and treat or prevent complications. Therefore, MDT may be an important approach to effectively treat mobility limitation, and it is crucial to initiate treatment of mobility limitation in a hospital setting.

Many studies have assessed univariate interventions for treating patients with mobility limitation in the community or hospitals, identifying positive effects, such as reduced fall rates and mortality. However, no research has examined the effects of the MDT approach in treating mobility limitation using RCTs in the hospital setting. Therefore, this study aims to evaluate the effectiveness of MDT in patients with mobility limitation in a hospital setting. This study will provide novel ideas for treating mobility limitation, consequently reducing adverse prognosis, shortening the length of hospital stay, decreasing hospitalization costs, and improving the quality of life of older adults.

## Data Availability

The data will be used under license for the current study, and further inquiries can be directed to the corresponding author/s.

## Abbreviations

ADL: Activities of Daily Living
FIST: Function Impairment Screening Tool
GDS-15: Geriatric Depression Scale
MDT: Multidisciplinary Team
MMSE: Mini-Mental State Examination
MNA-SF: Mini Nutritional Assessment short-form
RCT: Randomized Controlled Trial
SF-12: Short Form − 12
SPPB: Short-Physical Performance Battery
